# A model six-month workshop for developing systematic review protocols at teaching hospitals: action research and scholarly productivity

**DOI:** 10.1186/s12909-021-02538-6

**Published:** 2021-02-10

**Authors:** Hiraku Tsujimoto, Yuki Kataoka, Yukihito Sato, Masahiro Banno, Emi Tsujino-Tsujimoto, Yukiyoshi Sumi, Ryuichi Sada, Takashi Fujiwara, Yoichi Ohtake, Junji Kumasawa, Haruki Imura, Yoshinobu Matsuda, Ryuhei So, Tomoko Kagawa, Takashi Yoshioka, Yu Uneno, Hiroyuki Nagano, Mai Akazawa, Takunori Hozumi, Yasushi Tsujimoto

**Affiliations:** 1 Hospital Care Research Unit, Hyogo Prefectural Amagasaki General Medical Center, Amagasaki, Japan; 2 Systematic Review Workshop Peer Support Group (SRWS-PSG), Osaka, Japan; 3 Section of Clinical Epidemiology, Department of Community Medicine, Graduate School of Medicine, Kyoto University, Kyoto, Japan; 4 Department of Cardiology, Hyogo Prefectural Amagasaki General Medical Center, Amagasaki, Japan; 5 Department of Psychiatry, Seichiryo Hospital, Nagoya, Japan; 6 Department of Psychiatry, Nagoya University Graduate School of Medicine, Nagoya, Japan; 7 Department of General Internal Medicine, Kanai Hospital, Kyoto, Japan; 8 Department of Psychiatry, Shiga University of Medical Science, Otsu, Japan; 9 Department of General Internal Medicine, Kameda Medical Center, Kamogawa, Japan; 10 Department of Otolaryngology Neck and Surgery, Kurashiki Central Hospital, Kurashiki, Japan; 11 Department of Internal Medicine, Itami Seifu Hospital, Itami, Japan; 12 Department of Critical Care Medicine, Sakai City Medical Center, Sakai, Japan; 13 Department of Infectious Diseases, Rakuwakai Otowa Hospital, Kyoto, Japan; 14 Department of Psychosomatic Internal Medicine, National Hospital Organization Kinki-Chuo Chest Medical Center, Sakai, Japan; 15 Department of Health Promotion and Human Behavior, Kyoto University Graduate School of Medicine / School of Public Health, Kyoto, Japan; 16 Department of Respiratory Medicine, National Hospital Organization Kinki-Chuo Chest Medical Center, Sakai, Japan; 17 Department of Healthcare Epidemiology, Kyoto University Graduate School of Medicine / School of Public Health, Kyoto, Japan; 18 Center for Innovative Research for Communities and Clinical Excellence (CiRC2LE), Fukushima Medical University, Fukushima, Japan; 19 Department of Therapeutic Oncology, Graduate School of Medicine, Kyoto University, Kyoto, Japan; 20 Department of Healthcare Economics and Quality Management, Graduate School of Medicine, Kyoto University, Kyoto, Japan; 21 Department of Anesthesiology, Shiga University of Medical Science, Otsu, Japan; 22 Department of Critical Care Medicine, Aichi Children’s Health and Medical Center, Ohbu, Japan; 23 Department of Nephrology and Dialysis, Kyoritsu Hospital, Kawanishi, Japan

**Keywords:** Systematic reviews, Action research, Scholarly activity, Continuing professional development

## Abstract

**Background:**

Research engagement contributes to the improvement of patient care. A systematic review is a suitable first scholarly activity because it entails summarization of publicly available data and usually requires neither rigorous ethical review nor research funding.

**Methods:**

This study aimed to develop a model workshop for healthcare staff to acquire skills in creating systematic review protocols based on their own clinical questions at teaching hospitals. We used an action research method to create a model workshop at four hospitals in Japan from April 2015 to March 2017. To improve the program, we solicited reflections using participant questionnaires for each lecture and examined the quality of homework submitted by participants after each lecture. We administered a revised final version of the workshop at five hospitals from April 2016 to March 2017. We evaluated the participants’ scholarly productivity related to these workshops. The observation period was a minimum of 2 years following the workshops.

**Results:**

Most participants had never developed a formal clinical research protocol and voluntarily participated in the workshop. The action research was developed and implemented at nine teaching hospitals in Japan, including one university hospital. The study developed a model nine-step workshop curriculum: 1) Research question development, 2) Search strategy development, 3) Search strategy brush-up, 4) Exclusion and inclusion criteria development, 5) Risk of bias assessment planning, 6) Meta-analysis planning, 7) Subgroup and sensitivity analysis planning, 8) Planning the presentation of results, and 9) Presentation protocols. A total of 233 participants, including medical doctors and other health professionals, produced 414 research questions. Seventy-nine participants (34%) completed the workshop, and 47 review teams accomplished systematic review protocols. The participants published 13 peer-reviewed articles as a result of the workshop.

**Conclusions:**

We developed a structured scholarly productive model workshop for healthcare staff working at hospitals. We found healthcare staff with clinical subspecialties were able to develop an unexpectedly high number of research questions through this workshop. Medical teachers at hospitals with prior systematic review experience could teach how to develop systematic review protocols using this model. Further research is needed to increase the academic productivity of such workshops.

**Trial registration:**

UMIN (https://www.umin.ac.jp/ctr/), UMIN000017107 (4/15/2015), UMIN000025580 (1/10/2017).

**Supplementary Information:**

The online version contains supplementary material available at 10.1186/s12909-021-02538-6.

## Background

Research engagement is generally believed to contribute to the improvement of patient care [1, 2]. Residency programs in several countries include support for residents in their scholarly activities [3]. However, these programs may not involve adequate supervision and appropriate training in research methods [4, 5]. Systematic reviews (SRs) in medicine are a type of research that attempts to collate all empirical evidence to answer a specific research question [6]. The research method is a summarization of publicly available electronic patient data and usually requires neither rigorous ethical review nor research funding. This is an established and widely used method [7], allowing even research novices to perform SRs for their first clinical research projects.

Quality SRs require guidance and support of experienced researchers; however, these opportunities are rarely available for healthcare staff in their first SR. The first step in conducting an SR is to structure clinical questions for the research. Vague clinical questions often set too wide of a scope, requiring support from a senior with prior research experience to ensure the appropriateness of clinical questions. Unlike narrative literature reviews, SRs a priori specify eligibility criteria to be reviewed [7]. Authors of SRs usually consider eligibility criteria based on both clinical questions and specification of the types of studies that have addressed these questions [7]. Another feature of SRs is a comprehensive literature review to achieve unbiased knowledge syntheses [8]. If a modification of search strategies is required, the review authors are forced to repeat the entire time-consuming literature review process. Therefore, such a repetition can be avoided with a review of SR search strategies before running them. Protocol preparation for SRs requires an analysis of pre-specification of outcomes. Changes in SR outcomes observed in published SRs may lead to a biased interpretation of SR results [9, 10]. Therefore, step-by-step peer review of the protocols is essential for producing quality SRs. Although several universities and organizations have developed massive open online courses (MOOCs) for SRs [11–13], MOOCs involve large groups of people and render providing step-by-step feedback from teachers and advice tailored to the participants’ level of understanding a major challenge [14]. Furthermore, MOOCs are generally known for their high dropout rates [14–16].

In this study, we developed a model continuous workshop (WS) utilizing an action research method to develop SR protocols with step-by-step peer-review. We subsequently evaluated its scholarly productivity.

## Methods

We used an action research method to create a model WS and implemented it at nine teaching hospitals in Japan. The institutional ethics committees of each hospital approved our research. Action research is a research method that aims at both taking and creating knowledge or theory relating to that action [17–19]. The action research process involves a cyclical process of (1) planning, (2) implementing the plan (action), (3) observation, and (4) critical and self-critical reflection on the results of (1–3) and making decisions for the next cycle of (1–4) to improve the action. As such, the model WS was developed through a cyclical progress in four phases: planning, action, observation, and reflection [17]. The reflection phase after each lecture focused on attempting to improve both the next lecture for the same participants and a lecture on the same topic in the next hospital setting. The lectures were given by three teachers who were physicians with prior SR experience and one teacher who had been one of the participants of the third WS and completed an SR through the WS.

### Phase 1: planning

The goal of the WS was for healthcare staff to learn how to answer their own clinical questions through SRs. We recruited participants from each of the nine hospitals. Two teachers first constructed an overall framework for the WS based on prior experience with writing SRs, the graduate school syllabus at Kyoto University Graduate School of Medicine/School of Public Health, and an introductory textbook for SRs [7].

### Phase 2: action

We held the first WS from April to August 2015 at Hyogo Prefectural Amagasaki General Medical Center (HPAGMC) with 21 healthcare staff participants and held the second WS from October 2015 to February 2016 at Tenri Hospital with 14 healthcare staff participants. We held the third WS from April 2016 to September 2016 at Shiga University of Medical Science Hospital with 30 healthcare staff participants, including medical students and held the fourth WS from October 2016 to March 2017 at Kameda General Medical Center with 45 healthcare staff participants.

### Phase 3: observation

After each lecture, participants developed their SR protocols as their homework based on the lecture they took part in. We then assessed the quality of their protocols in terms of whether the study could actually be conducted in a reproduceable way and with reference to the Preferred Reporting Items for Systematic Review and Meta-Analysis Protocols (PRISMA-P) [20]. We also administered an open-response evaluation questionnaire and a Likert scale questionnaire (see Supplementary Table 1, Additional file 1) to participants after each lecture and developed a list of common questions and mistakes.

### Phase 4: reflection

Immediately after each lecture, we held a formal debriefing to evaluate the lecture and the homework. We read the questionnaire responses and discussed curriculum improvement, and thenmodified the subsequent lecture. All modifications were shared among the core members (HT, YK, and YT) and the external institutional members (ETT, YS2, and RS1) by email and approved through an interactive process until consensus was reached.

Finally, we discussed the framework of the model WS sessions in August 2016 for external validation. We conducted a WS using the revised final version of the curriculum at three hospitals (number of beds) in Japan: Sakai City Medical Center (487), National Hospital Organization Kinki-Chuo Chest Medical Center (385), Amagasaki Iryoseikyo Hospital (199). In addition, teachers who had not previously lectured in previous WSs independently conducted the WS at Kurashiki Central Hospital (1166) and Seirei Mikatahara General Hospital (934). Two years after the last WS, we analyzed the scholarly productivity related to the WS. The following terms were used to describe the participants’ outcomes with the following definitions:

- Research questions: a structured approach for framing questions that uses five components—the patient population or the disease being addressed, the interventions or exposure, the comparator group, the outcome or endpoint, and the study design chosen [7, 21]
- Protocol: a document that presents an explicit plan for a systematic review [20]
- Systematic review: an ﻿attempt to collate all relevant evidence that fits pre-specified eligibility criteria to answer a specific research question ﻿

The key characteristics of a systematic review are (1) a clear objective with an reproducible methodology, (2) a comprehensive search, (3) an assessment of the validity of the findings, and (4) systematic presentation and synthesis [20].

The observation period was a minimum of 2 years following completion of the workshop because a previous study showed that the median time from protocol registration to publication was over 1 year [22].

## Results

The first WS included seven interactive lecture-series from April 2015 to July 2015 at HPAGMC. Participants were mainly medical doctors, but other healthcare professionals were also present (Table 1) and voluntarily participated in the workshop. This section discusses our results under three main headings: structure of the final model WS (Table 2), quantitative data of each WS, and themes identified from the observations and reflections. The differences between the initial and final models are outlined in Table 3.

**Table 1:** Participant demographics

	HPAGMC	TH	SUMH	KMC	KCH	AISH	SGMC&KCCMC	SMGH	Total
Physician	14	11	13	39	15	23	31	18	164
Pharmacist	3	0	3	0	2	0	11	0	19
OT/PT/ST	0	2	7	6	1	3	0	0	19
Nurse	3	0	2	0	1	0	8	0	14
Dietician/CP/MLT	1	0	2	0	6	0	0	0	9
Medical student	0	0	3	0	2	0	0	0	5
Medical librarian	0	1	2	0	0	0	0	0	3
Total	21	14	32	45	27	26	50	18	233

*OT* occupational therapist, *PT* physical therapist, *ST* speech-language-hearing therapist, *CP* clinical psychologist, *MLT* medical lab technologist, *HPAGMC* Hyogo Prefectural Amagasaki General Medical Center, *TH* Tenri Hospital, *SUMH* Shiga University of Medical Science Hospital, *KMC* Kameda Medical Center, *KCH* Kurashiki Central Hospital, *AISH* Amagasaki Iryo Seikyo Hospital, *SGMC* Sakai City General Medical Center, *KCCMC* Kinki-Chuo Chest Medical Center, *SMGH* Seirei Mikatahara General Hospital

**Table 2:** The final WS model

No.	Agenda/Topic	Objective	Due at the next lecture
1	• Introduction to the workshop • What kind of RQs can and cannot be addressed by SRs • Preliminary search of existing SRs relevant to RQs • How to structure RQs for SR • Time management for research	• Understand what SR is • Finding existing SRs	• Prepare some RQs
2	• How to develop a search strategy using the Cochrane Library	• Develop a comprehensive search strategy	• Preliminary search using structured search formulas and controlled words
3	• How to develop a search strategy using MEDLINE • How to use reference manager	• Develop a comprehensive search strategy	• Develop a comprehensive search strategy
4	• How to develop inclusion and exclusion criteria • Understanding review teams	• Develop inclusion and exclusion criteria for the review	• Develop inclusion and exclusion criteria
5	• How to assess risk of bias • How to use search formulas from previous SRs	• Understand how to assess risk of bias	• Experience risk-of-bias assessment with examples
6	• How to perform meta-analyses • How to deal with heterogeneity • How to deal with publication bias	• Understand how to deal with publication bias • Understand how to perform data synthesis	• Perform meta-analysis using a prepared dataset • Revise drafted protocol sections
7	• How to synthesize results in a narrative	• Understand how to present SR results	• Revise drafted protocol sections
8	• Understanding the principles of GRADE • How to prepare the Summary of Findings table • How to register the protocol in a systematic review registry	• Understand how to assess the quality of evidence using the GRADE approach • Understand how to present SR results	• Revise drafted protocol sections
9	• Presentation day for protocols		• Revise drafted protocol sections

*RQ* Research question, *SR* Systematic review, *GRADE* Grading of Recommendations Assessment, Development, and Evaluation

**Table 3:** Differences between initial and final models

	Initial model	Final model
Target	Everyone	Everyone, especially medical staff with a subspecialty
Method of teaching	Traditional classroom	Flipped classroom
Model SR protocol	No	Distributed on the first day of the workshop
Time management advice	No	Yes
Review team members	Maximum possible	Two or three members who meet frequently
Finding previous SR	No	Brief way to find previous SRs that cover RQ
Intervals between each lecture	Two weeks	Three weeks
Feedback platform	Email based	Shared web cloud-based message board

*RQ* Research question, *SR* Systematic review

### Structure of the final model WS

We set specific objectives for each lecture, with the teaching method including short lectures and homework-based discussions (Table 2). Participants were expected to develop a part of their SR protocols based on the previous lecture as homework. They submitted the homework through an online platform (Google Forms, https://www.google.com/intl/ja_jp/forms/about/). We gave feedback using the online platform within 48 h of the submission and accepted resubmissions to ensure that participants would not lose the research rhythm [14]. All participants could see other participants’ homework and feedback and could use it as a reference for their own homework. Learning and practicing the items in Table 2 was essential for creating a research protocol that complies with the items in PRISMA-P. The process of action research and evaluation of the participant-created protocols improved the initial model.

### Themes identified from observations and reflections

During the observation and reflection phases, we carried out the following evaluations and then made modifications to develop the final model WS described above.

#### Participants’ readiness

Some participants had difficulty coming up with research questions due to a lack of clinical experience and knowledge. In such cases, the research scope tended to be overly broad and difficult to handle as an SR. On the other hand, participants with clinical subspecialties, such as specialized nurses and specialists in physical therapists, occupational therapists, or speech-language-hearing therapists, had many viable research questions. Therefore, we particularly encouraged clinical staff with a subspecialty to join the WS.

Most participants had never developed a formal clinical research protocol before, so some participants found it difficult to grasp the overall picture of the SR protocol and the connection between each lecture. Ultimately, some of these participants quit the WS if they found it challenging to develop a single section of the protocol, particularly the search formula development. To remedy this, we provided a model SR protocol in the first lecture and explained how each section of the protocol is related to each lecture.

Some participants also dropped out after finding existing SRs that covered their research questions. Although there is no way to find existing SRs without conducting an SR, previous SRs can be found quickly by using clinical practice guidelines and the Cochrane library (https://www.cochranelibrary.com/). To address this, we added a short lecture on finding existing SRs in the first meeting of the WS starting with the second hospital.

#### Time management for research activity

Most participants were full-time healthcare staff and had no time allocated for research within their work schedule. Unfortunately, lack of time was a consistent reason for participants dropping out of the WS. To address this, we provided an estimated time commitment for each homework assignment in advance and incorporated a time-management skill lecture in the first meeting of the WS [14]. In this lecture, we emphasized the importance of setting aside scheduled research time at least once a week. In addition, we extended the intervals between WSs from 2 to 3 weeks to allow more time for homework assignments.

In addition, as most participants were healthcare professionals, their priority was their patients, sometimes making it difficult for them to attend a scheduled lecture. To remedy this, we implemented flipped classrooms [23], where participants watched lectures online (https://youtu.be/OT5C9vbNYAc), collaborated in online discussions, and then carried out research activities as homework. Implementing flipped classrooms allowed participants to skip some WS meetings if necessary. To facilitate participation in the meetings without having questions to ask, some participants requested that the lecture be both provided on video and given at the WS meeting. However, it was not possible to do both because of limited time available for the meetings in the evening after work.

#### Research team

We identified one successful case where busy junior residents were able to create an SR protocol. We believe the reason for this success could be the support of their team members. The members of this research team met every day in the hospital ward. As one participant in the WS at HPAGMC observed, “Every time they met, they reminded each other that they had a research project to tackle together.”

On the other hand, teams with too many members had trouble finding time when all members could meet. These issues were not considered in the initial planning; therefore, at the first meeting, we facilitated participants in setting up their review team with two or up to three members who could meet frequently [24]. We encouraged choosing participants who could meet every day to facilitate quick peer support for their task.

Since attending meetings was not required, review teams were not always able to meet. To reduce communication obstacles and to foster camaraderie among the WS participants, we changed the feedback platform from email-based to a shared web cloud-based message board that every participant could read and participate in.

### Scholarly productivity

A total of 233 participants produced 414 research questions. Seventy-nine participants (34%) finished the WS. They wrote 47 protocols adhering to current standards [20]. If the participants were interested, we offered support in implementing the protocols and with publication. As a result, they published 13 peer-reviewed articles. We searched PubMed for SRs associated with affiliations of the hospitals that conducted the workshops in the 3 years before and after the workshops and found a threefold increase in the number of publications from before to after the WSs (Supplementary Tables 2, 3, Additional file 1). This is a larger number compared to about a 1.6 times increase for SRs associated with Japanese affiliations, suggesting an indirect effect of the WSs’ implementation. One of the early participants ultimately became a teacher of this WS. Although unanticipated at the time of planning, due to the success of this WS, some teachers were invited to several clinical practice guideline working groups, where experts teach SR research methods. In addition, the WS helped teachers develop a meta-epidemiological perspective on the process of SR creation, enabling them to publish 47 papers inspired by the WS as of May 2020 (Supplementary Table 4, Additional file 1; Supplementary data, Additional file 2).

## Discussion

During this project, we used action research methods to build and refine a model WS on developing SR protocols. We conducted the WS at 9 hospitals for over 200 healthcare staff, improved the quality of the lectures based on feedback and self-reflection, and derived a workable model. We found that healthcare staff were able to develop an unexpectedly high number of clinical questions through this WS.

The WS model has several strengths. Since the lecture had already been video recorded for the flipped classrooms, the WS was easily transferable between institutions in Japan. We also made the lecture materials available to everyone via the Internet (https://drive.google.com/drive/folders/1IfO9rNbGf2F_Tc7iJammVwl69vXxJ8P_?usp=sharing). The workshop eventually had a form of recruitment that could be easily transformed into a location-independent format, and we were able to start a crowdfunded online salon (https://community.camp-fire.jp/projects/view/187310). In the first year, this led to 252 participants, 16 protocols, and 1 paper accepted. Although we did not anticipate this at the beginning, the salon has become a form of learning that aligns well with the “stay-at-home” situation of the COVID-19 pandemic [25]. In addition, the model requires only one or two tutors, a small room with a projector, internet access, desks, chairs, and no standardized participants; therefore, it can be easily used in locations where educational resources are limited. Furthermore, the model allows participants easy access to peer reviewers through a peer discussion of their protocols. The participants may have played a role similar to that of teaching assistants in the previously reported university course, leading to the success of this workshop [26]. A notable success story is when one of the participants (MB), who was new to SR during the third WS, became a competent tutor in the eighth and ninth WSs, contributing greatly to the WS’s sustainability. Our WS also had a higher completion rate (34%) than most MOOCs, which generally have a completion rate of less than 13% [15]. We believe these results demonstrate that, rather than providing uniform information to all learners, teachers must strive to support individual learners [16, 27]. Given the participants’ readiness in this WS, this appears to be particularly important for learners at hospitals. Such individually catered education cannot be provided by MOOCs. In addition, we believe our online-based quick feedback system helped participants manage their time and maintain a rhythm of engagement in the study.

There were three main limitations to this WS model. First, the number of participants was limited to about 20–30 learners per teacher at a time, whereas MOOCs usually accept an unlimited number of applicants. Second, the proposed model WS was only validated in a limited number of institutions in one country in one language. We encourage further validation in different languages and settings. Finally, due to scheduling constraints, we could not perform a formal focus group session for the groups that dropped out or those who successfully published a paper. We believe that future research comparing the results of such focus groups would improve the academic productivity of the WS.

## Conclusion

We developed a structured model for interactive lecture-series WSs at hospitals. We showed that medical teachers in hospitals with prior SR experience could use this model to teach health professionals how to develop SR protocols. Further research is needed to increase the academic productivity of our model.

## Supplementary Information

**Additional file 1: Supplementary Table 1.** Questionnaire for participants. A questionnaire given to workshop participants. **Supplementary Table 2.** Number of SRs in PubMed affiliated with the hospitals for three years before and after the WS. PubMed search results for SRs three years before and after the WS. **Supplementary Table 3.** PubMed search details related to Supplementary Table 2. Search details related to Table 2. **Supplementary Table 4.** Issues covered by participants' SRs. Research topics for participants’ SRs

**Additional file 2: Supplementary data.** Publications related to the systematic review workshop since 2015.4

## Data Availability

The data used during the current study are available from the corresponding authors on reasonable request.

## Abbreviations

WS: Workshop
SR: Systematic review
MOOCs: Massive open online courses
HPAGMC: Hyogo Prefectural Amagasaki General Medical Center
