# Supplementary material for: A model six-month workshop for developing systematic review protocols at teaching hospitals: action research and scholarly productivity
Source: BMC Med Educ. 2021 Feb 10;21:98. doi: 10.1186/s12909-021-02538-6 (PMC7875449; doi:10.1186/s12909-021-02538-6)
Supplement: Supplementary file 2 — Additional file 2: Supplementary data. Publications related to the systematic review workshop since 2015.4 [file 12909_2021_2538_MOESM2_ESM.docx]

Supplementary data: Publications related to the systematic review workshop since 2015.4

**From participants:**

1.Banno M, Harada Y, Taniguchi M, Tobita R, Tsujimoto H, Tsujimoto Y, Kataoka Y, Noda A. Exercise can improve sleep quality: a systematic review and meta-analysis. PeerJ. 2018;6:e5172.

2.Taito S, Taito M, Banno M, Tsujimoto H, Kataoka Y, Tsujimoto Y. Rehabilitation for patients with sepsis: A systematic review and meta-analysis. Lopez-Delgado JC, editor. PLoS One. 2018 Jul 26;13(7):e0201292.

3.Majima K, Hirata N, Muraki Y, Effect of adding right colon retroflexion to one forward-view examination during colonoscopy compared with two forward-view examinations: a systematic review and meta-analysis. Gastroenterological Endoscopy. 2019;61(1): p25-35

4.Taito M, Taito S, Banno M, Fujiwara T, Okamura H, Tsujimoto H, Kataoka Y, Tsujimoto Y. Voice rehabilitation for laryngeal cancer after radiotherapy: a systematic review and meta-analysis. Eur Arch Oto-Rhino-Laryngology. 2019 May 4

5.Yasuda H, Kondo N, Yamamoto R, Asami S, Abe T, Tsujimoto H, Tsujimoto Y, Kataoka Y. Monitoring of gastric residual volume during enteral nutrition. Cochrane Database Syst Rev. 2019 May 14

6.Taito S, Yamauchi K, Tsujimoto Y, Banno M, Tsujimoto H, Kataoka Y. Does enhanced physical rehabilitation following intensive care unit discharge improve outcomes in patients who received mechanical ventilation? A systematic review and meta-analysis. BMJ Open. 2019 Jun 9;9(6):e026075.

7.Taito S, Kawai Y, Liu K, Ariie T, Tsujimoto Y, Banno M, Kataoka Y. Diarrhea and patient outcomes in the intensive care unit: Systematic review and meta-analysis. J Crit Care. 2019 Jun

8.Miki S, Tsujimoto Y, Shimada H, Tsujimoto H, Yasuda H, Kataoka Y, Fujii T. Non-pharmacological interventions for preventing clotting of extracorporeal circuits during continuous renal replacement therapy. Cochrane Database Syst Rev. 2019 May

9.Masuda F, Nakajima S, Miyazaki T, Yoshida K, Tsugawa S, Wada M, et al. Motor cortex excitability and inhibitory imbalance in autism spectrum disorder assessed with transcranial magnetic stimulation: a systematic review. Transl Psychiatry. 2019;9(1):110.

10.Kohmura K, Banno M, Tsujimoto H, Tsujimoto Y, Kataoka Y. Humour-based interventions for people with schizophrenia. Cochrane Database Syst Rev. 2019 Jul 4

11.Shiroshita A, Tanaka Y, Nakashima K, Furukawa Y, Kataoka Y. Diagnostic accuracy of specific IgG antibodies for bird fancier’s lung: a systematic review and meta-analysis. Ann Transl Med. 2019 Nov;7(22):655–655.

12.Muroi D, Ohtera S, Kataoka Y, Banno M, Tsujimoto Y, Tsujimoto H, Higuchi T. Obstacle avoidance training for individuals with stroke: a systematic review and meta-analysis. BMJ Open. 2019 Dec 16;9(12):e028873.

13.Namekawa M, Tsujimoto Y, Banno M, Kataoka Y, Tsujimoto H, Inaba Y. Videolaryngoscopy for transesophageal echocardiography probe insertion: a systematic review and meta ‑ analysis of randomized controlled trials. J Anesth. 2020

**From instructors:**

1. Fujii T, Ganeko R, Kataoka Y, Featherstone R, Bagshaw SM, Furukawa TA. Polymyxin B-immobilised haemoperfusion and mortality in critically ill patients with sepsis/septic shock: a protocol for a systematic review and meta-analysis. BMJ Open. 2016;6(11):e012908.

2.Tsujimoto H, Tsujimoto Y, Nakata Y, Fujii T, Akazawa M, Kataoka Y. Pharmacological interventions for preventing clotting of extracorporeal circuits during continuous renal replacement therapy (Protocol). Tsujimoto H, editor. Cochrane Database Syst Rev. 2016 Dec 10

3.Ichimasa K, Kudo S, Miyachi H, Kouyama Y, Ishida F, Toshiyuki B, Baba T, Katagiri A, Wakamura K, Hayashi T, Hisayuki T, Kudo T, Misawa M, Mori Y, Matsudaira S, Kimura Y, Kataoka Y. Patient gender as a factor associated with lymph node metastasis in T1 colorectal cancer: A systematic review and meta-analysis. Mol Clin Oncol. 2017 Feb 22;P0906.

4.Tsujimoto Y, Tsujimoto H, Kataoka Y, Kimachi M, Shimizu S, Ikenoue T, et al. Majority of systematic reviews published in high-impact journals neglected to register the protocols: a meta-epidemiological study. J Clin Epidemiol. Elsevier Inc.; 2017 Feb;0(0).

5.Tsujimoto Y, Tsujimoto H, Nakata Y, Kataoka Y, Kimachi M, Shimizu S, et al. Dialysate temperature reduction for intradialytic hypotension for people with chronic kidney disease requiring haemodialysis (Protocol). Cochrane Database Syst Rev. 2017;

6.Tsujimoto H, Tsujimoto Y, Nakata Y, Akazawa M, Kataoka Y. Ultrasonography for confirmation of gastric tube placement. Cochrane Database of Systematic Reviews. Chichester, UK: John Wiley & Sons, Ltd; 2016.

7.Shinohara K, Aoki T, So R, Tsujimoto Y, Suganuma AM, Kise M, et al. Influence of overstated abstract conclusions on clinicians: a web-based randomised controlled trial. BMJ Open. 2017;7(12):e018355.

8.Kumasawa J, Shimizu S, Nakano Y, Kataoka Y, Tsujimoto H, Tsujimoto Y. Doppler trans-thoracic echocardiography for detection of pulmonary hypertension in adults (protocol). Cochrane Database Syst Rev. 2017 Sep 26;(9).

9.Tsujimoto Y, Tsutsumi Y, Kataoka Y, Tsujimoto H, Yamamoto Y, Papola D, et al. Association between statistical significance and time to publication among systematic reviews: a study protocol for a meta-epidemiological investigation. BMJ Open. 2017;7(10):e018856.

10.Tsujimoto H, Tsujimoto Y, Kataoka Y. Unpublished systematic reviews and financial support: a meta-epidemiological study. BMC Res Notes. 2017 Dec 6;10(1):703.

11.Fujii T, Ganeko R, Kataoka Y, Furukawa TA, Featherstone R, Doi K, et al. Polymyxin B-immobilized hemoperfusion and mortality in critically ill adult patients with sepsis/septic shock: a systematic review with meta-analysis and trial sequential analysis. Intensive Care Med. Springer Berlin Heidelberg; 2017;

12.Yamazaki H, So R, Matsuoka K, Kobayashi T, Shinzaki S, Matsuura M, Okabayashi S, Kataoka Y, Tsujimoto Y, Furukawa TA, Watanabe N. Certolizumab pegol for induction of remission in Crohn’s disease (protocol). Cochrane Database Syst Rev. 2017 Dec 6;(12).

13.So R, Shinohara K, Aoki T, Tsujimoto Y, Suganuma AM, Furukawa TA. Effect of recruitment methods on response rate in a web-based study for primary care physicians: a factorial randomized controlled trial. Journal of Medical Internet Research 2018 Feb 8;20(2):e28

14.Ikegaki S, Matsumoto H, Kataoka Y, Hirano K, Tsujimoto H, Nakata Y, Katsura M. Third-line systemic chemotherapy for small cell lung cancer. Cochrane Database Syst Rev. 2018 May 7

15.Kataoka Y, Luo Y, Chaimani A, Onishi A, Kimachi M, Tsujimoto Y, Murad MH, Li T, Cipriani A, Furukawa TA. Cumulative network-meta-analyses, practice guidelines and actual prescriptions of drug treatments for postmenopausal osteoporosis: a study protocol for cumulative network meta-analyses and meta-epidemiological study. BMJ Open. 2018;8(12):e023218.

16. Luo Y, Chaimani A, Kataoka Y, Ostinelli EG, Ogawa Y, Cipriani A, Salanti G, Furukawa TA. Evidence synthesis, practice guidelines and real-world prescriptions of new generation antidepressants in the treatment of depression: a protocol for cumulative network meta-analyses and meta-epidemiological study. BMJ Open. 2018;8(12):e023222.

17.Imakita T, Matsumoto H, Hirano K, Morisawa T, Sakurai A, Kataoka Y. Impact on prognosis of rebiopsy in advanced non-small cell lung cancer patients after epidermal growth factor receptor-tyrosine kinase inhibitor treatment : a systematic review. 2019;1–5.

18.Banno M, Tsujimoto Y, Kataoka Y. Reporting quality of the Delphi technique in reporting guidelines: a protocol for a systematic analysis of the EQUATOR Network Library. BMJ Open. 2019 Apr 3;9(4):e024942.

19.Watanabe D, Hayashi H, Kataoka Y, Hashimoto T, Ichimasa K, Miyachi H, Tanaka S, Toyonaga T. Efficacy and safety of endoscopic submucosal dissection for non-ampullary duodenal polyps: A systematic review and meta-analysis. Dig Liver Dis. 2019 Apr

20.Niihata K, Shimizu S, Tsujimoto Y, Ikenoue T, Fukuhara S, Fukuma S. Variations and characteristics of quality indicators for maintenance hemodialysis patients: a systematic review. Health Sci Rep 2018 Sep 5;1(11):e89.

21.Tsujimoto Y, Aoki T, Shinohara K, So R, Suganuma AM, Kimachi M, Yamamoto Y, Furukawa TA. Physician characteristics associated with proper assessment of overstated conclusions in research abstracts: A secondary analysis of a randomized controlled trial. PLoS One. 2019 Jan 25;14(1):e0211206

22.Yajima N, Tsujimoto Y, Fukuma S, Sada K, Shimizu S, Niihata K, Takahashi R, Asano Y, Azuma T, Kameda H, Kuwana M, Kohsaka H, Sugiura-Ogasawara M, Suzuki K, Takeuchi T, Tanaka Y, Tamura N, Matsui T, Mimori T, Fukuhara S, Atsumi T. The development of quality indicators for systemic lupus erythematosus using electronic health data: a modified RAND appropriateness method. Modern Rheumatology.2019. (in press)

23.Tsutsumi Y, Tsutsumi I, Tsujimoto Y, Takahashi S, Tsuchiya A, Miyakoshi C, Fukuma S, Furukawa TA. Hyperbaric oxygen therapy for persistent post-concussion syndrome following mild traumatic brain injury (Protocol). Cochrane Database of Systematic Reviews 2017, Issue 7. Art. No.: CD012727.

24.Omae K, Tsujimoto Y, Honda M, Kondo T, Tanabe K, Fukuhara S, Furukawa T. Comparative Efficacy and Safety of Bone-modifying Agents for the Treatment of Bone Metastases in Patients with Advanced Renal Cell Carcinoma: A Systematic Review and Meta-analysis. Oncotarget 2017 18;8(40):68890-68898.

25.Tsujimoto Y, Tsutsumi Y, Kataoka Y, Tsujimoto H, Yamamoto Y, Papola D, Guyatt GH, Fukuhara S, Furukawa TA. Statistical significance did not affect time to publication in non-Cochrane systematic reviews: a meta-epidemiological study. J Clin Epidemiol. 2019 Jul

26.Tsujimoto Y, Tsujimoto H, Nakata Y, Kataoka Y, Kimachi M, Shimizu S, Ikenoue T, Fukuma S, Yamamoto Y, Fukuhara S. Dialysate temperature reduction for intradialytic hypotension for people with chronic kidney disease requiring haemodialysis. Cochrane Database Syst Rev. 2019 Jul 5

27.Sakurada T, Ueda A, Komukai D, Uchiyama K, Tsujimoto Y, Yuasa H, Ryuzaki M, Ito Y, Tomo M, Nakamoto H. Outcomes after peritoneal dialysis catheter placement by laparoscopic surgery versus open surgery: systematic review and meta-analysis. Renal Replacement Therapy 2019; 5: 37.

28.Maruyama Y, Higuchi C, Io H, Wakabayashi K, Tsujimoto H, Tsujimoto Y, Yuasa H, Ryuzaki M, Ito Y, Nakamoto H. Comparison of peritoneal dialysis and hemodialysis as first renal replacement therapy in patients with end-stage renal disease and diabetes: a systematic review. Renal Replacement Therapy 2019 (in press).

29.Ito M, Saka Y, Kuroki Y, Yasuda K, Tsujimoto H, Tsujimoto Y, Yuasa H, Ryuzaki M, Ito Y, Nakamoto H. Assessment of the effect of angiotensin-converting enzyme inhibitors and angiotensin receptor blockers in peritoneal dialysis patients: a systematic review and meta-analysis on clinical trials. Renal Replacement Therapy 2019 5:42

30.Yamazaki H, So R, Matsuoka K, Kobayashi T, Shinzaki S, Matsuura M, et al. Certolizumab pegol for induction of remission in Crohn’s disease. Cochrane Database Syst Rev. 2019;(8).

31.Banno M, Tsujimoto Y, Kataoka Y. Studies registered in non-ClinicalTrials.gov accounted for an increasing proportion of protocol registrations in medical research. J Clin Epidemiol. 2019 Sep

32.Omae K, Kataoka Y, Tsujimoto Y, Tsutsumi Y, Fukuhara S, Furukawa T. Publication statuses of clinical trials supporting immune checkpoint inhibitors recently approved by the United States Food and Drug Administration: A meta-epidemiological investigation. BMC Cancer. 2019 Oct 24;19(1):998.

33.Tsujimoto Y, Fujii T, Onishi A, Omae K, Luo Y, Imai H, Takahashi S, Itaya T, Pinson C, Nevitt SJ, Furukawa TA. No consistent evidence of data availability bias existed in recent individual participant data meta-analyses: A meta-epidemiological study. J Clin Epidemiol. 2019 Oct 22. pii: S0895-4356(19)30473-1.

34.Kotani Y, Kataoka Y, Izawa J, Fujioka S, Yoshida T, Kumasawa J, Kwong JS. High versus low blood pressure targets for cardiac surgery with cardiopulmonary bypass. Cochrane Database Syst Rev. 2019 Nov 29

35.Tsujimoto Y, Tsutsumi Y, Ohnishi T, Kimachi M, Yamamoto Y, Fukuhara S. Low pre-dialysis plasma calculated osmolality is associated with higher all-cause mortality: The Japanese Dialysis Outcomes and Practice Patterns Study (J-DOPPS). Nephron 2019 (in press)

36.Fujii T, Belletti A, Carr A, Furukawa TA, Luethi N, Putzu A, Sartini C, Salanti G, Tsujimoto Y, Udy AA, Young PJ, Bellomo R. Vitamin C therapy for patients with sepsis or septic shock: a protocol for a systematic review and a network meta-analysis. BMJ Open 2019; 9: e033458.

37.Kataoka Y, Sakurai A, Mori H, Yoshida H, Nakano Y, Fujii K, Matsushita R, Suzuki R, Shiraishi R, Takada T. A Workshop on Writing Letters to the Editor. MedEdPublish. 2020;9(1).

38.Luo Y, Kataoka Y, Ostinelli EG, Cipriani A, Furukawa TA. National Prescription Patterns of Antidepressants in the Treatment of Adults With Major Depression in the US Between 1996 and 2015: A Population Representative Survey Based Analysis. Front Psychiatry. 2020 Feb 14;11.

39.Kataoka Y, Luo Y, Chaimani A, Onishi A, Kimachi M, Tsujimoto Y, Murad MH, Li T, Cipriani A, Furukawa TA. Cumulative network meta-analyses, practice guidelines, and actual prescriptions for postmenopausal osteoporosis: a meta-epidemiological study. Arch Osteoporos. 2020 Dec 23;15(1):21.

40.Tsujimoto H, Tsujimoto Y, Nakata Y, Fujii T, Takahashi S, Akazawa M, Kataoka Y. Pharmacological interventions for preventing clotting of extracorporeal circuits during continuous renal replacement therapy. Cochrane Database Syst Rev. 2020 Mar 13

41.Banno M, Tsujimoto Y, Kataoka Y. The majority of reporting guidelines are not developed with the Delphi method: A systematic review of reporting guidelines. J Clin Epidemiol. 2020

42.Taito S, Kataoka Y. Assessment of publication trends of systematic reviews and randomized controlled trials of rehabilitation. Ann Phys Rehabil Med. 2020 Apr 18

43.Obata Y, Murashima M, Toda N, Yamamoto S, Tsujimoto Y, Tsujimoto Y, Tsujimoto H, Yuasa H, Ryuzaki M, Ito Y, Tomo T, Nakamoto H. Topical application of mupirocin to exit sites in patients on peritoneal dialysis: a systematic review and meta-analysis of randomized controlled trials. Renal Replacement Therapy 2020 6:12.

44.Morimoto K, Terawaki H, Washida N, Kasai T, Tsujimoto Y, Yuasa H, Ryuzaki M, Ito Y, Tomo M, Nakamoto H. The impact of intraperitoneal antibiotic administration in patients with peritoneal dialysis-related peritonitis: systematic review and meta-analysis. Renal Replacement Therapy 2020 6:19

45.Tsutsumi Y, Tsujimoto Y, Takahashi S, Tsuchiya A, Fukuma S, Yamamoto Y, Fukuhara S. Accuracy of aortic dissection detection risk score alone or with D-dimer: A systematic review and meta-analysis. Eur Heart J Acute Cardiovasc Care. 2020 Jan 23:2048872620901831.

46.Watanabe J, Park D, Kakehi E, Inoue K, Ishikawa S, Kataoka Y. Efficacy and safety of the starting position during colonoscopy: a systematic review and meta-analysis. Endoscopy International Open. 2020 (in press).

47.Luo Y, Chaimani A, Furukawa TA, Kataoka Y, Ogawa Y, Cipriani A, Salanti G. Visualizing the Evolution of Evidence: Cumulative Network Meta-Analyses of New Generation Antidepressants in the Last 40 Years. Res Synth Methods. 2020 Apr 30;50(6):jrsm.1413.
